# Person-Centered Care for Family Caregivers: Co-Designing an Education Program for the Healthcare Workforce

**DOI:** 10.1093/geroni/igab046.1265

**Published:** 2021-12-17

**Authors:** Jasneet Parmar, Sharon Anderson, Cheryl Pollard, Lesley Charles, Bonnie Dobbs, Myles Leslie, Cecelia Marion, Gwen McGhan

**Affiliations:** 1 Department of Family Medicine, University of Alberta, Edmonton, Alberta, Canada; 2 Faculty of Medicine & Dentistry University of Alberta, Edmonton, Alberta, Canada; 3 McEwan University, Edmonton, Alberta, Canada; 4 University of Alberta, Edmonton, Alberta, Canada; 5 University of Calgary, Calgary, Alberta, Canada; 6 Covenant Health, St. Albert, AB, Alberta, Canada

## Abstract

Background: Research recommends the healthcare workforce receive competency-based education to support family-caregivers [FCGs}. typically, education has been directed at FCG’s to increase their care skills rather that at healthcare providers to provide person-centered care to FCGs. Objectives: We present the co-design process used to create a competency-based education program for the healthcare workforce that ensures a person-centered focus on FCGs and introduce our Health Workforce Caregiver-Centered Care Education. Approach: Co-design is the act of creating with stakeholders to ensure useable results that meet stakeholder’s needs. We began by coining the concept “caregiver-centered care,” defined as a collaborative working relationship between families and healthcare providers aimed at supporting FCGs in their caregiving role, decisions about care management, and advocacy. From this definition we co-designed, then validated the Caregiver-Centered Care Competency Framework in a Delphi Process. Stakeholders (n= 101) including FCGs, providers, policy makers, community organizations, researchers, and educational designers then used effective practices for health workforce education to co-design the ‘foundational’ level of a Caregiver Centered Care education. Results: Teaching and learning resources include six competency-aligned educational modules with videos and interactive exercises that encourage reflection. With the COVID-19 pandemic, we moved the education online (caregivercare.ca). In the first four months online, 815healthcare providers completed the education. We continue to use mixed methods to evaluate the Caregiver-Centered Care Education, for acceptability and effectiveness, in five care contexts (primary, acute, home, supportive living, long-term care). Conclusion: We expect that our education will support caregiver-centered care in all healthcare settings.

